# Single-base-resolution methylome of giant panda’s brain, liver and pancreatic tissue

**DOI:** 10.7717/peerj.7847

**Published:** 2019-10-17

**Authors:** Jianying Ren, Fujun Shen, Liang Zhang, Jie Sun, Miao Yang, Mingyu Yang, Rong Hou, Bisong Yue, Xiuyue Zhang

**Affiliations:** 1 Key Laboratory of Bio-resources and Eco-environment (Ministry of Education), College of Life Sciences, Sichuan University, Chengdu, China; 2 Sichuan Key Laboratory of Conservation Biology for Endangered Wildlife, Chengdu Research Base of Giant Panda Breeding, Chengdu, China

**Keywords:** Giant panda, WGBS, Low/high methylation, DMR, Tissue-specific function

## Abstract

The giant panda (*Ailuropoda melanoleuca*) is one of the most endangered mammals, and its conservation has significant ecosystem and cultural service value. Cytosine DNA methylation (5mC) is a stable epigenetic modification to the genome and has multiple functions such as gene regulation. However, DNA methylome of giant panda and its function have not been reported as of yet. Bisulfite sequencing was performed on a 4-day-old male giant panda’s brain, liver and pancreatic tissues. We found that the whole genome methylation level was about 0.05% based on reads normalization and mitochondrial DNA was not methylated. Three tissues showed similar methylation tendency in the protein-coding genes of their genomes, but the brain genome had a higher count of methylated genes. We obtained 467 and 1,013 different methylation regions (DMR) genes in brain vs. pancreas and liver, while only 260 DMR genes were obtained in liver vs pancreas. Some lncRNA were also DMR genes, indicating that methylation may affect biological processes by regulating other epigenetic factors. Gene ontology and Kyoto Encyclopedia of Genes and Genomes analysis indicated that low methylated promoter, high methylated promoter and DMR genes were enriched at some important and tissue-specific items and pathways, like neurogenesis, metabolism and immunity. DNA methylation may drive or maintain tissue specificity and organic functions and it could be a crucial regulating factor for the development of newborn cubs. Our study offers the first insight into giant panda’s DNA methylome, laying a foundation for further exploration of the giant panda’s epigenetics.

## Introduction

Epigenetics study heritable changes in gene expression that do not involve altering the DNA sequence (Fazzari & Greally, 2004). DNA methylation, which is one of the most studied epigenetic modifications, is common in animals, plants and even fungi. It has been proven to play important roles, like cell development, complex diseases, phenotype change and evolutionary process (Martienssen & Colot, 2001; Ziller et al., 2013). Basically, DNA methylation, which is the addition of a methyl group (CH3) covalently to cytosine, most commonly at the C5-methylcytosine (5mC), is transmitted with high fidelity over many cell generations through mitosis and transgenerational through meiosis (Bock & Lengauer, 2008; Heard & Martienssen, 2014). It is now recognized that DNA methylation, in concert with other regulators, is a major epigenetic factor influencing gene activities (Gibney & Nolan, 2010; Jaenisch & Bird, 2003).

Unique to Carnivora, giant pandas are specialized herbivores with an almost exclusive bamboo diet, but it retains a typical carnivore’s digestive tract. Moreover, giant panda possesses elusive reproductive traits such as giving birth to most altricial neonate (Jin et al., 2011; Li et al., 2010; Wei et al., 2015). All these features have attracted the extensive attention of geneticists and evolutionary biologists. Considering the function of DNA methylation in rapid adaptation to changing environment and heritable nature, it may also be reflected in some biological processes of giant panda (Baker-Andresen, Ratnu & Bredy, 2013; Dimond & Roberts, 2016; Heyn et al., 2013; Sevane, Martínez & Bruford, 2019). Meanwhile, it has been demonstrated that epigenetic mechanisms regulate gene expression as potential candidates to confer tissue-specific gene expression and the epigenetic alterations are also increasingly implicated in metastasis (Chatterjee et al., 2017; Mathers, Strathdee & Relton, 2010). The absence of comprehensive genome-wide profiling and functional analysis of DNA methylation has hindered our understanding of epigenetic regulation of the giant panda.

Earlier analyses on DNA methylation often focused on promoter regions or CpG islands (CGI) through microarray and hence only partial DNA methylation status was observed (Deaton & Bird, 2011; Jones, 2012; Martienssen & Colot, 2001). Currently, several methods falling into three major assay types are utilized to study DNA methylation. Among these assays, whole-genome bisulfite sequencing is the gold standard (Ziller et al., 2016). Differentiated cells develop a stable and unique DNA methylation pattern that regulates tissue-specific gene transcription (Moore, Le & Fan, 2013). The pancreas and liver tissues are involved in digestion and metabolism, and the brain has obviously multi-functional features, as its regulatory function to multiple organs, including digestive organ (D’Aldebert et al., 2009; Douris & Maratos–Flier, 2013; Myers & Olson, 2012; Peters et al., 2011). In this study, we performed bisulfite sequencing (BS-seq) on the three tissues of a 4-day-old captive giant panda simultaneously which is a critical period in the development of a giant panda. We surveyed the methylation status at single-base scale, calculated methylation level of promoter and conducted pairwise comparison. Our goal was to obtain the tissue-specific influence of DNA methylation on the newborn cub of giant panda.

## Materials and Methods

### Material and sequencing

Sample collection and utility protocols were approved by the Ethics Committee, College of Life Sciences, Sichuan University (Grant No: 20190506001). Our experimental procedures complied with the current laws on animal welfare and research in China. The brain, liver and pancreatic tissues were collected from a 4-day-old giant panda which was the progeny of “Aibang” (http://www.panda.org.cn/english/news/events/2014-08-20/4185.html). This newborn baby had been fed by his mother and subjected to 24-h onsite alternate care by the base nursery staff since his birth. Nothing abnormal about the cub was detected in terms of body temperature, urination, defecation and breast milk intake in the first 4 days after his birth, and his weight has increased from 145 g at birth to 172 g. The giant panda cub died due to asphyxia and all visceral organs appeared to be normal (http://www.panda.org.cn/english/news/news/2016-05-12/5649.html) (Peng et al., 2018). All methods were performed in accordance with the relevant guidelines and regulations by professional veterinarians. The fresh tissue samples were immediately stored at −80 °C. Genomic DNA of the tissues was extracted following the Qiagen DNeasy Blood & Tissue Kit. DNA purity was checked using the Nano Photometer spectrophotometer (IMPLEN, Westlake Village, CA, USA). DNA concentration was measured using Qubit DNA Assay Kit in Qubit 2.0 Flurometer (Life Technologies, Carlsbad, CA, USA). Library construction and sequencing was performed using the same protocols employed by a previous study (Xu et al., 2018).

### Raw reads QC and BS-seq data alignment

At first, the raw bisulfite-converted paired-end reads were quality checked using FastQC v.0.11.5 (Simon Andrews, http://www.bioinformatics.babraham.ac.uk/projects/fastqc/), adapter and low quality reads were trimmed by using Trim Galore! v.0.4.4 (Felix Krueger, http://www.bioinformatics.babraham.ac.uk/projects/trim_galore/) with the parameter -q 20 –length 20 and it was then rechecked using FastQC. Bismark v.0.18.2 was utilized to align the trimmed sequence reads to each of four bisulfite converted reference genomes, which were derived from the conversion of each strand and the corresponding complement using Bowtie2 v.2.3.2 with –N 1 --score-min L,0,-0.2 --ignore-quals --no-mixed --no-discordant --maxins 1000, with the other parameters set as default (Krueger & Andrews, 2011). After deduplication (Picard-tools v2.9.0-1-SNAPSHOT) and removing non-conversion bases, methylation was subsequently called for each covered cytosine and the summary statistics were calculated using the bismark_methylation_extractor script.

### Differential methylation analysis

The detection of different methylation regions (DMR) is a prerequisite for characterizing different epigenetic status. DMRs were predicted using metilene in de-novo mode among sites with at least 10× coverage. The metilene software uses a binary segmentation algorithm combined with a two-dimensional statistical test to calculate the DMR. Whether in the framework of international consortia with dozens of samples per group, or even without biological replicates, it produces highly significant and reliable results (Jühling et al., 2016). We then used the metilene_output.pl script to filter the output file using the criterion of at least 10 CpGs within a DMR and *q*-value at 0.05.

### Metaplot analysis

For metagene plot, gene body regions were divided proportionally with 20 bins. Upstream 2,000 bp or downstream 2,000 bp regions were divided into 20 bins (100 bp in each bin). The average methylation level of each bin was calculated for each gene and plotted by Python.

### GO and KEGG analyses

Gene ontology (GO) enrichment analysis of DMR-overlapping genes were performed using the R package clusterProfiler (v3.6.0) (Yu et al., 2012). GO terms with *q*-value less than 0.05 were considered statistically significant. The Kyoto Encyclopedia of Genes and Genomes (KEGG) analysis of DMR-overlapping genes uses KOBAS 3.0 (http://kobas.cbi.pku.edu.cn/) (Wu et al., 2006).

All other calculations and picture plotting in this study used in-house Python (v2.7.13) or R (R Core Team, 2018) scripts.

## Results

### DNA methylation whole-genome bisulfite sequencing, quality control and reads mapping

We performed whole genome BS-seq of brain, liver and pancreatic tissues obtained from the male giant panda. Approximately 988 million (64.5×) BS-seq raw reads were generated from brain, 1,079 million (70.4×) from liver and 703 million (45.9×) from pancreas (Ziller et al., 2015). The raw reads are available from the NCBI SRA browser (Bioproject accession number, PRJNA482275). The BS-seq conversion rate was 99.67%, 99.60% and 99.69%, respectively. Almost 99.99% of the raw reads passed the QC but with different read length.

Generally, DNA methylome studies are based on genome, hence, a better assembled genome and good annotation is important. To be prudent, we chose the NCBI Refseq edition (genome build accession: GCF_000004335.2), due to its annotation strategies and more extensive feature annotation, and the mapping efficiency was 50.4% (brain), 73.91% (liver) and 70.5% (pancreas) (Li et al., 2010). Vertebrate CGI are short interspersed DNA sequences that deviate significantly from the average genomic pattern by being CpG-rich and approximately 70% of annotated gene promoters are associated with a CGI (Deaton & Bird, 2011). We used newcpgplot, which is a part of EMBOSS toolkit (http://emboss.sourceforge.net/), to determine the genome’s CpG island with default parameters and got 72,965 CGI. We chose 1,000 bp upstream of the first exon as promoter and at least one CpG island within it is called high CpG content (HCG), otherwise, it is a low CpG content (LCG). We got 11,431 HCG and 3,351 LCG—with only 58.6% being HCG (75% in human) (Saxonov, Berg & Brutlag, 2006).

### 5mC calling and whole genome methylation level analysis

The whole genome methylation level is about 0.05% according to reads normalization (Figs. S1–S3). In the three tissues, about 249, 398 and 247 million paired-end reads are uniquely mapped. There are 14, 21 and 13 billion cytosine analyzed in total with 76.2%, 68.7% and 70.4% cytosines methylated in the CpG context. Due to breakage of the genome DNA randomly while building the sequencing library, we checked the genome coverage of the reads to reveal the coverage of reads. The result showed that about 80% cytosine loci were at least 5× coverage (Figs. S4–S6). In total, 95.24%, 96.44% and 94.77% cytosine sites in the reference genome were covered by sequencing datasets. All these results reflect that the library construction and sequencing is pretty good. The CpG methylation distribution in tissues follows a bimodal distribution (Figs. S7 and S8). This means that most CpGs are either fully methylated or fully unmethylated (Akalin et al., 2012).

There are 6,518 bp cytosines in the panda’s mitochondrial DNA (mtDNA). As an example, in brain and pancreas, 6,410 and 6,428 cytosine loci are detected in the BS-data but the mC was rarely discovered even at the 751× and 773× on average, correspondingly. We can infer that the cytosine loci in the giant panda’s mitochondrial genome are not methylated and might play any function just like in human beings (Hong et al., 2013). Nevertheless, it indicates good consistency with the non-conversion rate.

The proportion of reads that supported methylation of covering depth at a specific site was generally defined as the methyl cytosine methylation level. To better depict the whole genome methylation level, we constructed a genome, which has 21 pseudo chromosomes, through a concatenation operation of the 81,466 scaffolds (mitochondrial genome not included). We then calculated the methylation level of 1,000 bp window size and 1,000 bp step size to get the whole-genome scale view of the three tissues’ methylome (Figs. S9–S11). The low methylation level in the end of pseudo chromosome 21 may be caused by too many short length scaffolds and unplaced contigs. At the whole-genome scale, most parts of the genome share common methylation status. Methylated cytosine always functions in a continuous way, therefore we calculated the methylation level in CpG, CHG and CHH context at 5× coverage separately within 10,000 bp window and the methylation level of cytosine in CHG, CHH context (H = A, T or C) is almost close to 0 (Fig. S12).

**Figure 1:**
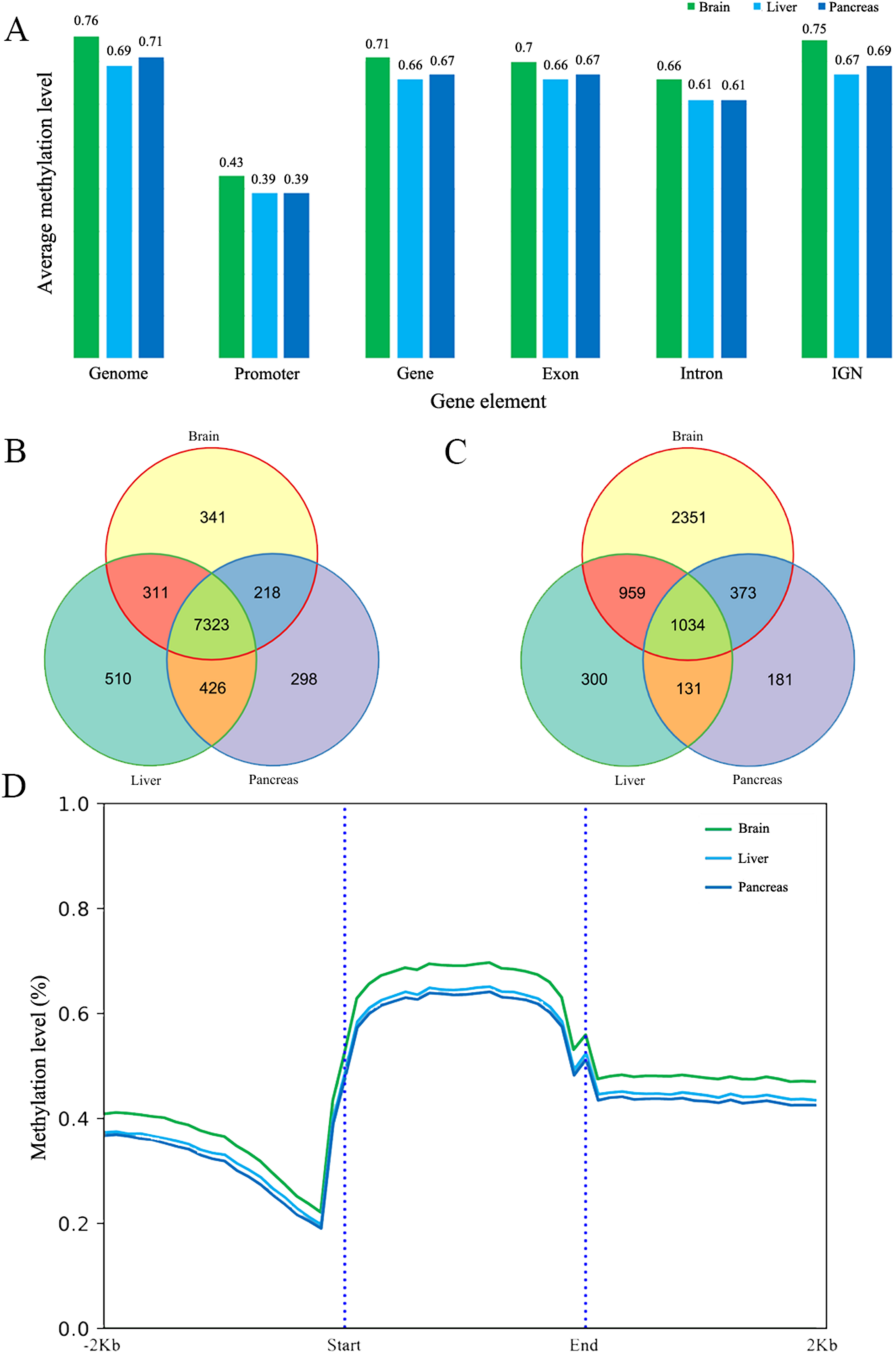
The DNA methylation level and patterns. (A) The mean methylation level in different gene elements including promoter, gene, exon, intron and intergenic region in three tissues; (B) and (C) The Venn diagram of low (B) and high (C) methylated promoter gene number of three tissues; (D) The methylation patterns diagram of protein-coding genes in brain, liver and pancreas. Start and end denotes the start and end locus of the protein-coding genes.

The methylated cytosines in different gene elements have different functions (Arechederra et al., 2018). We calculated the mean methylation level in promoters (we chose the upper 1,000 bp of the first exon as promoter according to other studies), exons, introns, genes and intergenic non-coding regions (IGN) (Akalin et al., 2012). The average methylation level of different elements is similar in the three tissues at 5× coverage. The promoter regions have the lowest methylation at 0.43, 0.39 and 0.39 respectively (Fig. 1A) (Saxonov, Berg & Brutlag, 2006). In addition to that, the methylation level in exons is slightly higher than that in intron and the IGN is a bit higher than in gene bodies. The methylation patterns of genic regions were similar to those of other species, showing increased CG methylation in gene bodies and flanking regions but reduced methylation in transcriptional start/end sites (Jones, 2012; Lister et al., 2009; Zhang et al., 2017). Meanwhile, CHH and CHG methylation was depleted in all regions (Jones, 2012). We also did the protein-coding genes’ metaplot analysis to better reflect the DNA methylation tendency across the gene body, upstream and downstream. CG methylation is increased in gene bodies and flanking regions but reduced in gene body start/end sites (Fig. 1D). Furthermore, CpG methylation in brain protein-coding gene is a little higher than liver and pancreas just as the mean methylation analysis.

### Low/High methylated promoter genes

DNA methylation in the promoter region is often associated with gene silencing (Deaton & Bird, 2011). We classified the promoter into low methylated promoter (LMP) and high methylated promoter (HMP), with these two categories using the mean methylation level 0.2 and 0.8 respectively (Eckhardt et al., 2006). Overall, the number of LMP genes is much more than HMP genes in the three tissues. It was found that the number of LMP is nearly equal in liver and pancreas whereas the brain possesses more methylated promoters than liver and pancreas (Figs. 1B and 1C). Based on the results of GO analysis, most of LMP genes’ GO items are about housekeeping functions but differently distributed in the three tissues studied. LMP genes in brain are specifically distributed at Wnt-related signaling pathway, DNA replication, regulation of axon genesis, pallium development, ncRNA processing and intracellular protein transport. The LMP genes between liver and pancreas are differentially distributed in peptide biosynthetic process, RNA metabolic process, DNA metabolic, regulation of cell cycle and protein transport and targeting. Additionally, HMP genes in brain are enriched at inflammatory and defense response. Based on KEGG analysis results, LMP genes are differently distributed at melanogenesis, Wnt signaling pathway, glioma and thermogenesis in brain. It’s worth pointing out that central melanin-concentrating hormone influences liver and adipose metabolism (Imbernon et al., 2013). The brain and pancreas have endocrine resistance and ErbB signaling pathway. Longevity regulation pathway is only distributed in liver. HMP genes in brain are enriched at cytokine-cytokine receptor interaction, platelet activation, JAK-STAT signaling pathway and Fc epsilon RI signaling pathway. However, in the liver it is enriched at necroptosis. Brain and pancreas both have JAK-STAT signaling pathway (Figs. S13 and S14). In combination with the tissues’ functions, all these GO and KEGG results reflect that DNA methylation, no matter low or high, might lead to common and different tissue functions even at this stage.

### Tissue-specific DMR profiling

**Figure 2:**
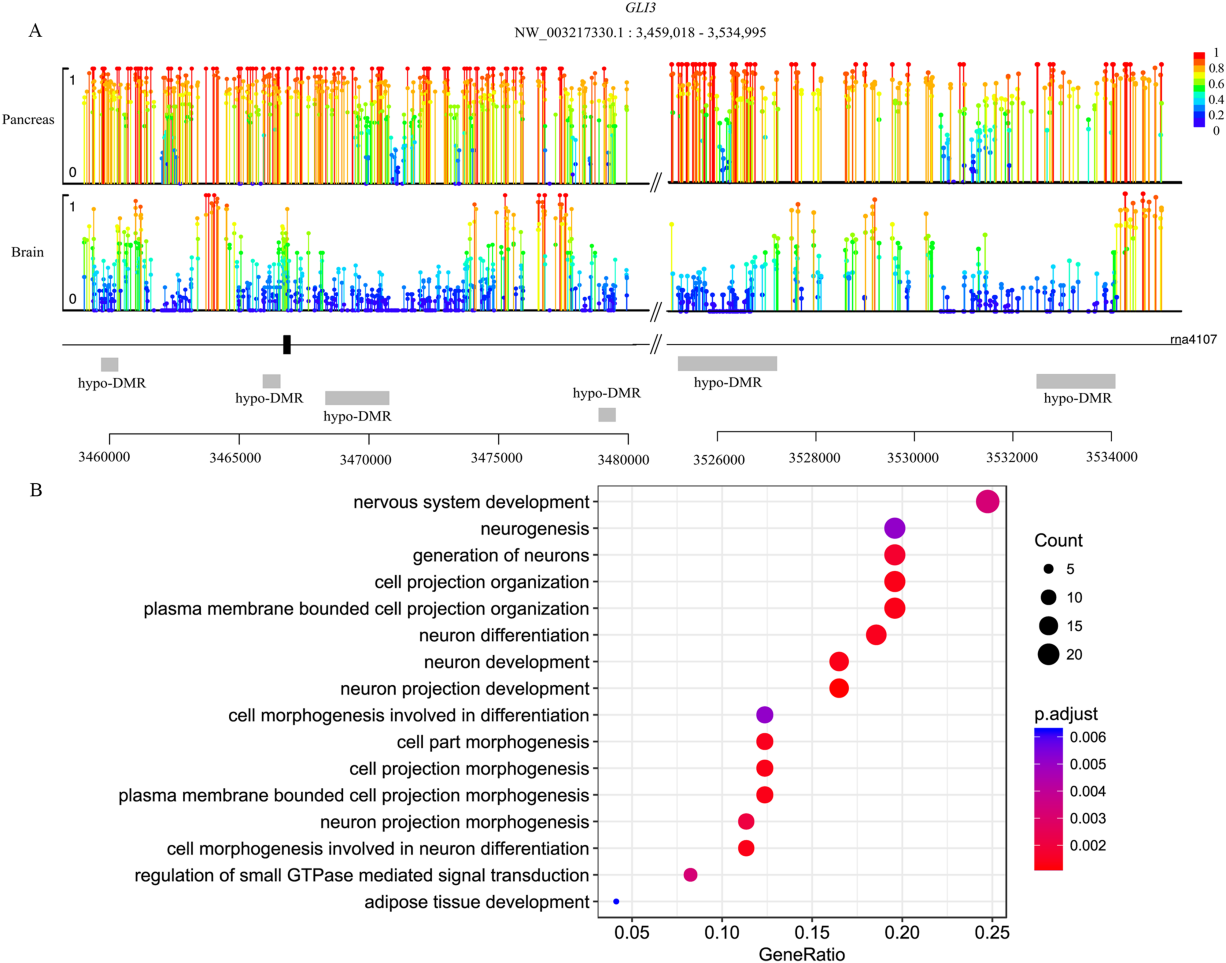
Lollipop plot of DNA methylation level in gene *GLI3* and DMR gene’s GO enrichment result in brain and pancreas comparison. (A) There are six DMRs that are all hypo with different length in *GLI3* gene. Mean methylation level (red to deep blue color), DMR status and DMR relative location are all labeled; (B) DMR genes’ GO enrichment result in the brain and pancreas comparison.

Genes are methylated either within their promoters or within their transcribed regions and gene methylation is highly correlated with transcription levels. Moreover, the function of gene body methylation (GbM) seems bigger than what we previously thought (Arechederra et al., 2018). GbM genes often compose the bulk of methylated genes within angiosperm genomes and are enriched for housekeeping functions (Bewick & Schmitz, 2017). Consequently, to eliminate uncertainties, we inspected the DMRs not only in promoters but also in whole gene bodies and got the DMR overlapping genes within upstream (1,000 bp), gene bodies and downstream (1,000 bp) (Akalin et al., 2012). With 10× coverage, a *p*-adjust value cutoff of 0.05 and at least 10 CpGs contained, we got 1,018 DMRs (421 hyper and 597 hypo) in brain vs pancreas (see Supplement File). On the intersection of at least 50% length of DMR, we got 467 DMRs overlapping genes. There are 67 genes including more than one DMRs and *GLI3* overlaps with six DMRs (Fig. 2A). Gene *GLI3* encodes C2H2-type zinc finger proteins that are subclass of the Gli family. They are characterized as DNA-binding transcription factors and function as both a repressor and activator of transcription which is specifically involved in the development of pancreas (Nielsen et al., 2008). Additionally, we found that DMRs not only overlap mRNA but also lncRNA (22), and even a tRNA (Nielsen et al., 2008; Zhao, Sun & Wang, 2016). Furthermore, it’s interesting that multi-DMRs also appear in lncRNA. Using the same criteria, we got 2,598 DMRs (755 hyper and 1,843 hypo) and 1,013 DMR genes in brain vs liver. There are 445 DMRs (371 hyper and 74 hypo) and 260 DMR genes in liver vs pancreas and the least DMR gene number probably indicates the tissue function similarity.

### Functional analysis of DMR overlapping genes

In the comparison of brain and pancreas, 23 genes show functions related to binding, seven genes are involved in activation/coactivation, 12 genes function as transcription factors and 11 genes are zinc finger proteins (see Supplement File supp.tables.xlsx sheet 4). These regulatory genes may cause extensive and complex consequences under DNA methylation. Different tissues manifest different energy requirements. Although DNA methylation is absent in mtDNA, we found that 10 DMR genes have a functional relationship with mitochondria. In addition, we found that *HDAC1*, *HDAC4* and *HDAC9* are also DMR genes. They are all histone deacetylases and responsible for the deacetylation of lysine residues on the N-terminal part of the core histones (H2A, H2B, H3 and H4). This means that DNA methylation may also have influences on histone (Zhang & Reinberg, 2001). Recent findings have suggested a more general role for the central nervous system in metabolic control (Myers & Olson, 2012). Neuronal systems can regulate energy intake, energy expenditure, and endogenous glucose production sense and respond to input from hormonal and nutrient-related signals (Schwartz & Daniel, 2005). Here, we found that some DMR genes are obesity genes or related to the former which have been studied by genomic and genome-wide association study (GWAS) methods, for example, *FTO, PCSK5*, *PCSK6*, *NTRK2*, *ABCC4*, *TXNIP* and *RPS6KA2* (Al Muftah et al., 2016; D’Angelo & Koiffmann, 2012; Dina et al., 2007; Milagro et al., 2013). The above mentioned genes are hypo-methylated in brain but hyper-methylated in liver and pancreas except *RPS6KA2* which is hyper-methylated in brain and pancreas while hypo-methylated in liver.

To gain further insight into the functional role of DMRs, we examined the enrichment of GO and KEGG pathways for the DMR-overlapping genes. In the comparison of brain and pancreas, these genes were significantly enriched in several developmental processes and signal transduction, including cell part morphogenesis (GO:0032990), adipose tissue development (GO:0060612), nervous system development (GO:0007399) and regulation of small GTPase mediated signal transduction (GO:0051056) (Fig. 2B). In KEGG pathway analysis, pathways that were identified are involved in many biological processes, like hormone regulation (aml04910, aml04922), metabolization (aml00061, aml00640), development (aml04392, aml04340) and brain functional pathways (aml04360, aml04530) (Table 1). Interestingly, the most significantly enriched pathway is the hippo signaling pathway (aml04392) which controls diverse aspects of cell proliferation, survival, and morphogenesis in eukaryotes and the core organization of these networks is conserved over a billion years of evolution. The cAMP signaling pathway is also significantly enriched. It’s a well-known signal pathway which uses cAMP as second messenger, responds to hormones and stimulates pivotal physiologic progresses including metabolism, secretion, gene transcription etc. In a nutshell, DNA methylation exhibits influence on these key pathways to show a more extensive and complex function. Although the whole genome methylation level is only 0.05, it influences key pathways and can have an extensive range of functions.

**Table 1:** Statistically significant enriched KEGG items of DMR genes in brain and pancreas comparison (Partial).

Term	ID	Input number	Background number	*p*-value
Hippo signaling pathway—multiple species	aml04392	5	29	0.000286698
cAMP signaling pathway	aml04024	12	197	0.000321699
Axon guidance	aml04360	11	173	0.000402346
Fatty acid biosynthesis	aml00061	3	12	0.002039626
Propanoate metabolism	aml00640	4	32	0.003433939
Hedgehog signaling pathway	aml04340	4	44	0.009636186
Glucagon signaling pathway	aml04922	6	99	0.010197961
Insulin signaling pathway	aml04910	7	138	0.013726705
Valine, leucine and isoleucine degradation	aml00280	4	51	0.015366193
Pancreatic secretion	aml04972	5	89	0.024286973
Pantothenate and CoA biosynthesis	aml00770	2	17	0.042526108

The GO enrichment results of brain and liver comparison shows that there are some enriched items which are related to immunity, for example, T cell differentiation (GO:0030217), alpha-beta T cell activation (GO:0046631) and alpha-beta T cell differentiation (GO:0046632) (Fig. S15). This means that DNA methylation influences the immunity functions of liver. This situation is also reflected in the KEGG enrichment analysis, for example, B cell receptor signaling pathway (aml04662), natural killer (NK) cell mediated cytotoxicity (aml04650) and Fc gamma R-mediated phagocytosis (aml104666) (Table 2). Additionally, the PI3K-Akt signaling pathway (aml04151), which can be activated by many types of cellular stimuli or toxic insults, and regulates fundamental cellular functions such as transcription, growth and survival, is also significantly enriched. Furthermore, we also found that some DMR genes are lncRNAs. A recent study of 5,641 lncRNA transcripts in rhesus macaques found a tight association with epigenetic markers and CGIs (Chen et al., 2015). The relationship of DNA methylation and lncRNA may be worth a more detailed study. In addition to this, DNA methylation also has different influences on liver and pancreas and this is reflected in GO and KEGG annotation, such as some metabolic processes, leukocyte activation and migration, T cell selection, PI3K-Akt signaling pathway and cAMP signaling pathway (Fig. S16 and S17).

**Table 2:** Enriched KEGG items of DMR genes in brain and liver comparison (Partial).

Term	ID	Inputnumber	Backgroundnumber	*p*-value
PI3K-Akt signaling pathway	aml04151	31	331	1.62891939132e-05
Hippo signaling pathway	aml04390	17	150	0.000172197945661
cAMP signaling pathway	aml04024	17	197	0.002799888959
Axon guidance	aml04360	22	173	3.93969253235e-06
Fatty acid elongation	aml00062	4	23	0.0168841307934
Propanoate metabolism	aml00640	4	32	0.0436637692321
Hedgehog signaling pathway	aml04340	8	44	0.000626603087941
Glucagon signaling pathway	aml04922	10	99	0.00747421215857
Valine, leucine and isoleucine degradation	aml00280	7	51	0.00555857761228
Pantothenate and CoA biosynthesis	aml00770	5	17	0.0010940947287
Natural killer cell mediated cytotoxicity	aml04650	10	92	0.0047030370925
Fc gamma R-mediated phagocytosis	aml04666	10	82	0.00221616313219
Fc epsilon RI signaling pathway	aml04664	7	65	0.017469871196
B cell receptor signaling pathway	aml04662	6	71	0.0668349466587

## Discussion

The various types of omics studies in the giant panda are helpful to understand the species at a system biological level and to unravel some biological mysteries—like the feed switch. The increasing number of recent studies have confirmed that epigenetics has a tight relationship with gene expression (Gibney & Nolan, 2010; Jaenisch & Bird, 2003). Moreover, studies have shown that adult tissues exhibit highly distinct genome-wide DNA methylation signatures, hypermethylation is highly tissue-specific and located within genes that are involved in tissue-specific processes (Byun et al., 2009; Rakyan et al., 2008; Slieker et al., 2015; Ziller et al., 2013).

Some fatty acids are major components of neuronal tissues, directly involved in processes of neuronal differentiation and survival and are considered to be crucial for brain growth and development (Glade & Smith, 2015; Salem et al., 2001; Zhang et al., 2016). Adipose tissue development (GO:0060612) and fatty acid biosynthesis (aml00061) are also significantly enriched, and some key genes are hypomethylated, like *ACACA* and *ACSL1*. Moreover, *ACACB*, which may be involved in the regulation of fatty acid oxidation rather than fatty acid biosynthesis, is hyper-methylated. All these findings indicate that fatty acids are prone to biosynthesis under the influence of DNA methylation for brain development. Pancreas is an organ, having both an endocrine and a digestive exocrine function. Some tissue-specific functions are enriched, such as pancreatic secretion, glucagon and insulin signaling pathways and fatty acid biosynthesis (Table 1; Fig. S13). The various functions of the liver are carried out by hepatocytes, like carbohydrate, lipid, protein, bile acid and bilirubin metabolism. In the comparison of brain and liver, our results show that the DMR genes are enriched not only in metabolism but also in some immunity related pathways, like NK cell mediated cytotoxicity (aml04650), Fc gamma R-mediated phagocytosis (aml04666) and Fc epsilon RI signaling pathway (aml04664). NK cells are lymphocytes of the innate immune system and equipped with various receptors whose engagement allows them to discriminate between target and nontarget cells (Cheent & Khakoo, 2009). Activating receptors bind ligands on the target cell surface and trigger NK cell activation and target cell lysis. The *CD48* gene which encodes a immunoglobulin-like receptor in the CD2 subfamily and is found on the surface of lymphocytes and other immune cells like *ICAM2* (which mediates adhesive interactions), *TNFSF10* (which induces apoptosis) are all important for NK-cell mediated clearance. They are all hypomethylated in liver, but the *FYN* gene, which encodes a membrane-associated tyrosine kinase that regulates immune response, was hypermethylation in liver. Considering the fact that *FYN* plays a role in many biological processes like axon guidance (hypomethylation in brain), we suspect that there may exist some other mechanism to make a compensation for its seemingly abnormal high methylation (Thude et al., 2015). In addition, studies have shown that normal postnatal hepatic development and maturation involves extensive epigenetic remodeling of the genome, adapting to the marked changes in the nutritional environment (Ehara et al., 2015; Cannon et al., 2016). In a word, it is easy to get a conclusion that DNA methylation has a prevalent influence on tissue-specific and organic functions. Unique to Carnivora, giant pandas are specialized herbivores. After weaning, giant pandas also have a large change in diet. To meet this alteration, whether existing epigenetic reprogramming for these important organs involved in digestion and metabolism, such as liver and pancreas, needs further study.

The giant panda not only gives birth to unusually immature and most altricial neonates with the smallest neonate-maternal weight ratio (about 1/1,000) among eutherian mammals, but it also exhibits the shortest known gestation among Ursidae (Zhu et al., 2001). In humans, some data strongly show that preterm infants frequently exhibit some abnormal features such as suboptimal nutrient intake in early postnatal life, growth failure within the neonatal intensive care unit (NICU), higher levels of insulin resistance, abnormal partitioning of fat deposition, with the brain being exquisitely vulnerable to undernutrition and later cognitive attainment being permanently affected by suboptimal nutrient intakes (Embleton, 2013). Therefore, the rapid development and large requirement of nutrition at the early postnatal stage might be vital for premature newborn cubs (Zhu et al., 2001). A prolonged transition time between colostrum and mature milk for the supply of immune protection and nutrition might be a compensatory strategy for unusually immature neonates (Griffiths et al., 2015). However, the rapid establishment of the neonates’ own physiological, metabolic and defensive systems should be a better guarantee to survive this critical period. For inducing these establishments, in the external environmental side, the miRNAs related to basic metabolism, neuron development and immunity were found to be horizontally transferred to the cubs from the giant panda’s breast milk (Ma et al., 2017). At individual itself side, based on the result of GO and KEGG analyses, these LMP genes have more items and their functions are mainly involved in basic metabolism and development, like cell cycle, DNA replication, translation, RNA transport, metabolic processes etc. According to the canonical view about the negative relationship of gene expression and DNA methylation, these LMP genes might be associated with the active expression of these genes and were also important for the development of the preterm newborn cubs. On the other hand, HMP genes have a smaller number of GO or KEGG items. All these results imply that the DNA methylation may be a crucial regulator for the development of giant panda newborn cubs.

## Conclusion

We have carried out, to our knowledge, the first DNA methylation study of giant panda. LMP genes might be associated with the active expression of these genes for organic functions and were important for the development of the delicate panda cubs. There were 1,018, 2,598 and 445 tissue specific DMRs discovered through three tissues’ pairwise comparison and it seems that similar functional tissues have the least number DMRs. GO and KEGG analyses results demonstrated that DNA methylation has a relationship with tissue-specific functions or biological processes and may be a crucial regulator for the development of giant panda newborn cubs. As the first step to elucidate the biological role of DNA methylation, this study reports the characteristics of DNA methylation of key growth stages and supplies important information for further insight into the regulatory functions of DNA methylation in the giant panda.

## Supplemental Information

10.7717/peerj.7847/supp-1 Supplemental Information 1 The reads methylation bias of the brain reads. (A) mbias plot of the original top reads in brain BS-seq data. (B) mbias plot of the original bottom reads in brain BS-seq data.

10.7717/peerj.7847/supp-2 Supplemental Information 2 The reads methylation bias of the liver reads. (A) mbias plot of the original top reads in liver BS-seq data. (B) mbias plot of the original bottom reads in liver BS-seq data.

10.7717/peerj.7847/supp-3 Supplemental Information 3 The reads methylation bias of the pancreas reads. (A) mbias plot of the original top reads in pancreas BS-seq data. (B) mbias plot of the original bottom reads in pancreas BS-seq data.

10.7717/peerj.7847/supp-4 Supplemental Information 4 Reverse cumulative plot coverage of the brain BS-seq reads. *X*-axis represents the coverage and *y*-axis represents the percentage of sites across genome. Cytosine at CG/CHG/CHH contexts are indicated by green/blue/red line.

10.7717/peerj.7847/supp-5 Supplemental Information 5 Reverse cumulative plot coverage of the liver BS-seq reads. *X*-axis represents the coverage and *y*-axis represents the percentage of sites across genome. Cytosine at CG/CHG/CHH contexts are indicated by green/blue/red line.

10.7717/peerj.7847/supp-6 Supplemental Information 6 Reverse cumulative plot coverage of the pancreas BS-seq reads. *X*-axis represents the coverage and *y*-axis represents the percentage of sites across genome. Cytosine at CG/CHG/CHH contexts are indicated by green/blue/red line.

10.7717/peerj.7847/supp-7 Supplemental Information 7 The histogram of methylated cytosine percentage at CpG context in brain. The numbers on bars denotes what percentage of locations are contained in that bin. Percent methylation histograms have two peaks on both ends, many locations with high methylation and some locations with low methylation.

10.7717/peerj.7847/supp-8 Supplemental Information 8 The histogram of methylated cytosine percentage at CpG context in pancreas. The numbers on bars denotes what percentage of locations are contained in that bin. Percent methylation histograms have two peaks on both ends, many locations with high methylation and some locations with low methylation. Note: The liver BS-seq data is too big for computation, hence, here only provide the histogram of methylated cytosine percentage at CpG context in brain and liver.

10.7717/peerj.7847/supp-9 Supplemental Information 9 The whole genome DNA methlation landscape of the brain. There are 21 pseudo chromosomes in total. The red point represents the relative higher mean methylation level, and the green represents the relative lower mean methylation level.

10.7717/peerj.7847/supp-10 Supplemental Information 10 The whole genome DNA methlation landscape of the pancreas. There are 21 pseudo chromosomes in total. The red point represents the relative higher mean methylation level, and the green represents the relative lower mean methylation level.

10.7717/peerj.7847/supp-11 Supplemental Information 11 The whole genome DNA methlation landscape of the liver. There are 21 pseudo chromosomes in total. The red point represents the relative higher mean methylation level, and the green represents the relative lower mean methylation level.

10.7717/peerj.7847/supp-12 Supplemental Information 12 The proportion of methylated cytosine in different cytosine context. The *X*-axis represents mean methylation levels binned in 10 increment of 10% (i.e., 0–10%, 10–20%), *y*-axis is the corresponding bin’s fraction.

10.7717/peerj.7847/supp-13 Supplemental Information 13 The GO enrichment of LMP/HMP genes. The *X*-axis is the LMP in brain, HMP in brain, LMP in liver and LMP in pancreas from left to right. The *y*-axis the GO items.

10.7717/peerj.7847/supp-14 Supplemental Information 14 The KEGG enrichment of LMP/HMP genes. The *X*-axis is the LMP in brain, HMP in brain, LMP in liver, HMP in liver, LMP in pancreas and HMP in pancreas from left to right. The *y*-axis the KEGG pathways.

10.7717/peerj.7847/supp-15 Supplemental Information 15 The GO enrichment of DMR genes of brain vs liver. The *X*-axis is gene ratio and the *y*-axis is the enriched GO items.

10.7717/peerj.7847/supp-16 Supplemental Information 16 The GO annotations of DMR genes of liver vs pancreas. The *X*-axis is gene number and the *y*-axis is the enriched GO items.

10.7717/peerj.7847/supp-17 Supplemental Information 17 The KEGG annotation of DMR genes of liver vs pancreas. The *X*-axis is gene number and the *y*-axis is the enriched KEGG pathways.

10.7717/peerj.7847/supp-18 Supplemental Information 18 DMR profile, LHP & HMP gene id.

10.7717/peerj.7847/supp-19 Supplemental Information 19 SRA accessions of the data at NCBI.

## Additional Information and Declarations

### Data Availability

The following information was supplied regarding data availability: The raw NGS data is available at NCBI Bioproject under accession number PRJNA482275.
