# Supplementary figures and images for: Single-base-resolution methylome of giant panda’s brain, liver and pancreatic tissue
Source: PeerJ. 2019 Oct 17;7:e7847. doi: 10.7717/peerj.7847 (PMC6800980; doi:10.7717/peerj.7847)

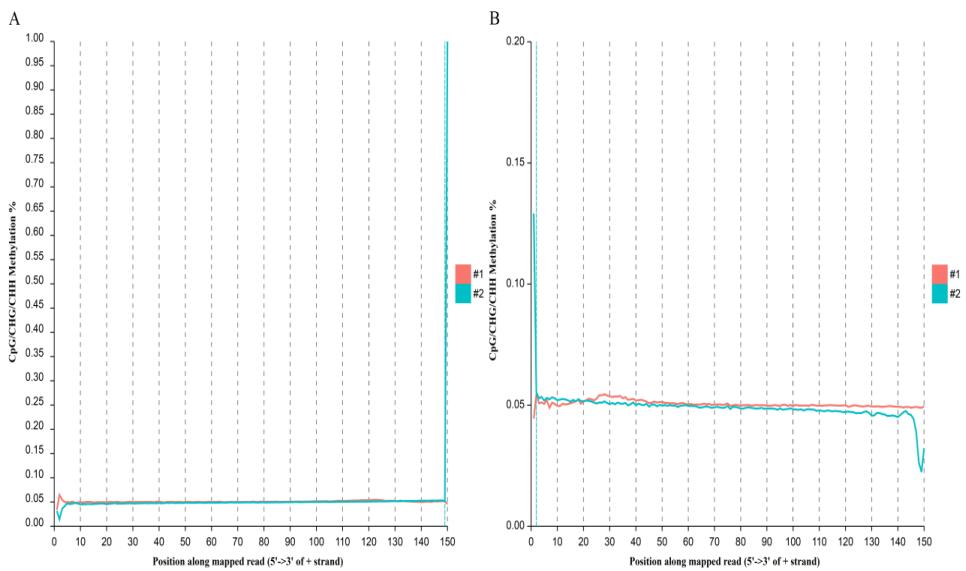

Supplement: Supplemental Information 1 — (A) mbias plot of the original top reads in brain BS-seq data. (B) mbias plot of the original bottom reads in brain BS-seq data. [file peerj-07-7847-s001.jpg]

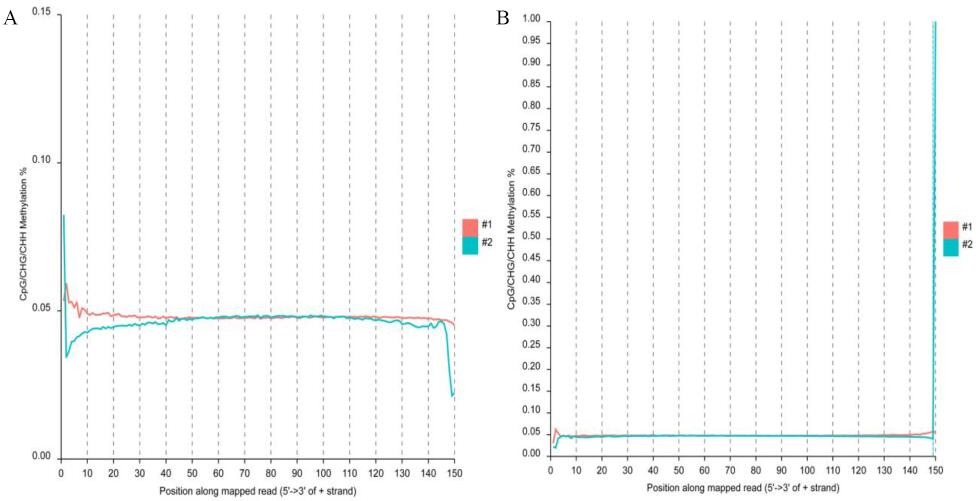

Supplement: Supplemental Information 2 — (A) mbias plot of the original top reads in liver BS-seq data. (B) mbias plot of the original bottom reads in liver BS-seq data. [file peerj-07-7847-s002.jpg]

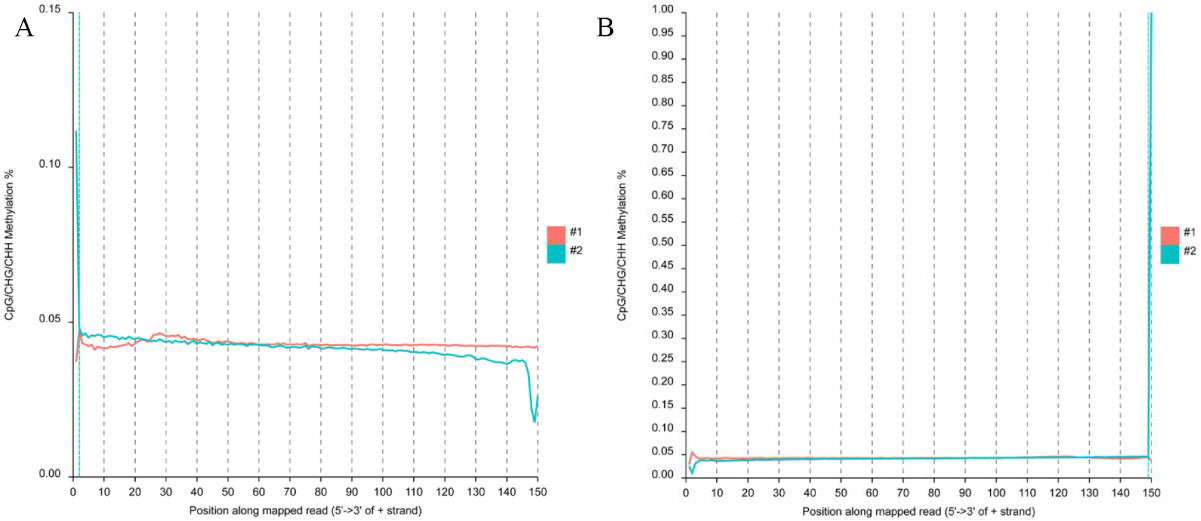

Supplement: Supplemental Information 3 — (A) mbias plot of the original top reads in pancreas BS-seq data. (B) mbias plot of the original bottom reads in pancreas BS-seq data. [file peerj-07-7847-s003.jpg]

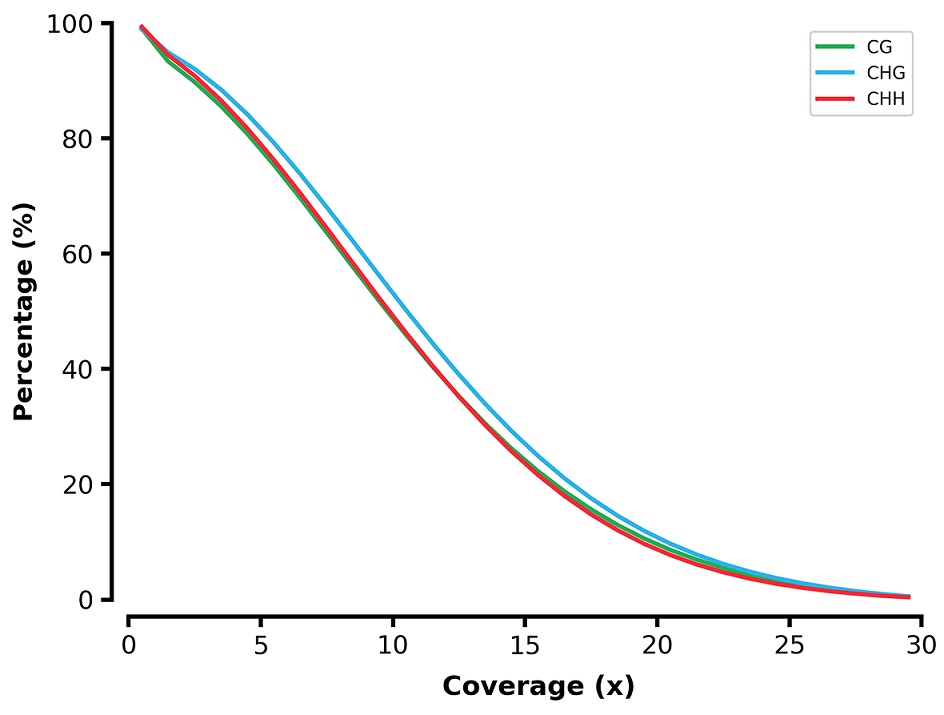

Supplement: Supplemental Information 4 — X-axis represents the coverage and y-axis represents the percentage of sites across genome. Cytosine at CG/CHG/CHH contexts are indicated by green/blue/red line. [file peerj-07-7847-s004.jpg]

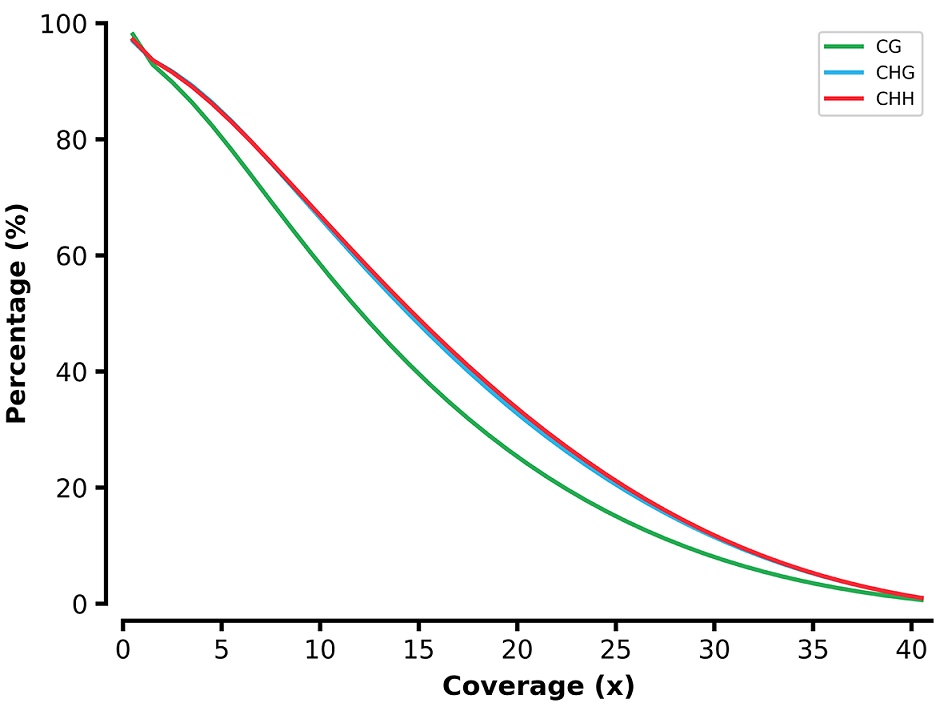

Supplement: Supplemental Information 5 — X-axis represents the coverage and y-axis represents the percentage of sites across genome. Cytosine at CG/CHG/CHH contexts are indicated by green/blue/red line. [file peerj-07-7847-s005.jpg]

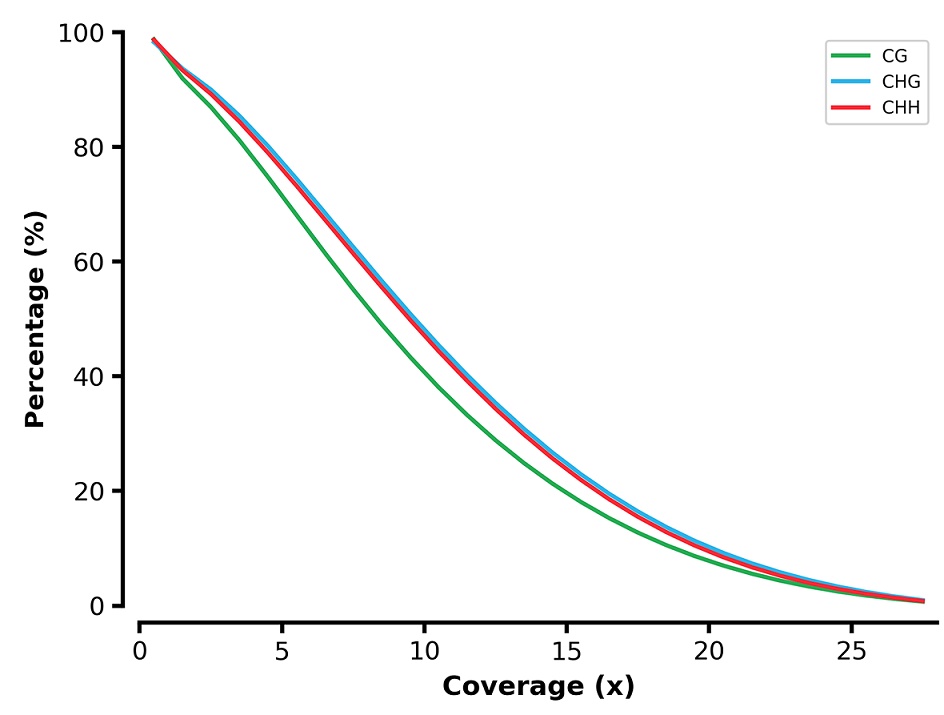

Supplement: Supplemental Information 6 — X-axis represents the coverage and y-axis represents the percentage of sites across genome. Cytosine at CG/CHG/CHH contexts are indicated by green/blue/red line. [file peerj-07-7847-s006.jpg]

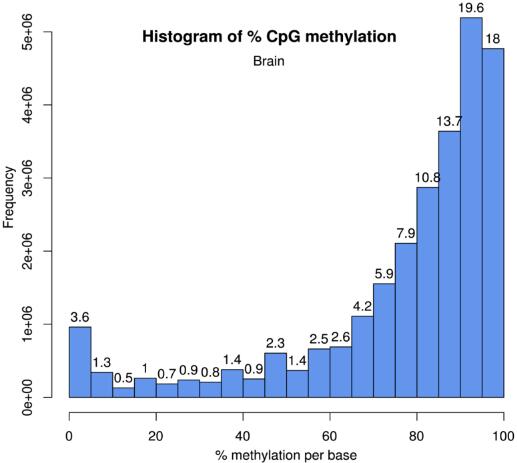

Supplement: Supplemental Information 7 — The numbers on bars denotes what percentage of locations are contained in that bin. Percent methylation histograms have two peaks on both ends, many locations with high methylation and some locations with low methylation. [file peerj-07-7847-s007.jpg]

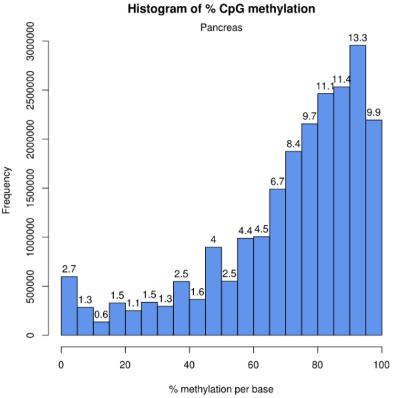

Supplement: Supplemental Information 8 — The numbers on bars denotes what percentage of locations are contained in that bin. Percent methylation histograms have two peaks on both ends, many locations with high methylation and some locations with low methylation. Note: The liver BS-seq data is too big for computation, hence, here only provide the histogram of methylated cytosine percentage at CpG context in brain and liver. [file peerj-07-7847-s008.jpg]

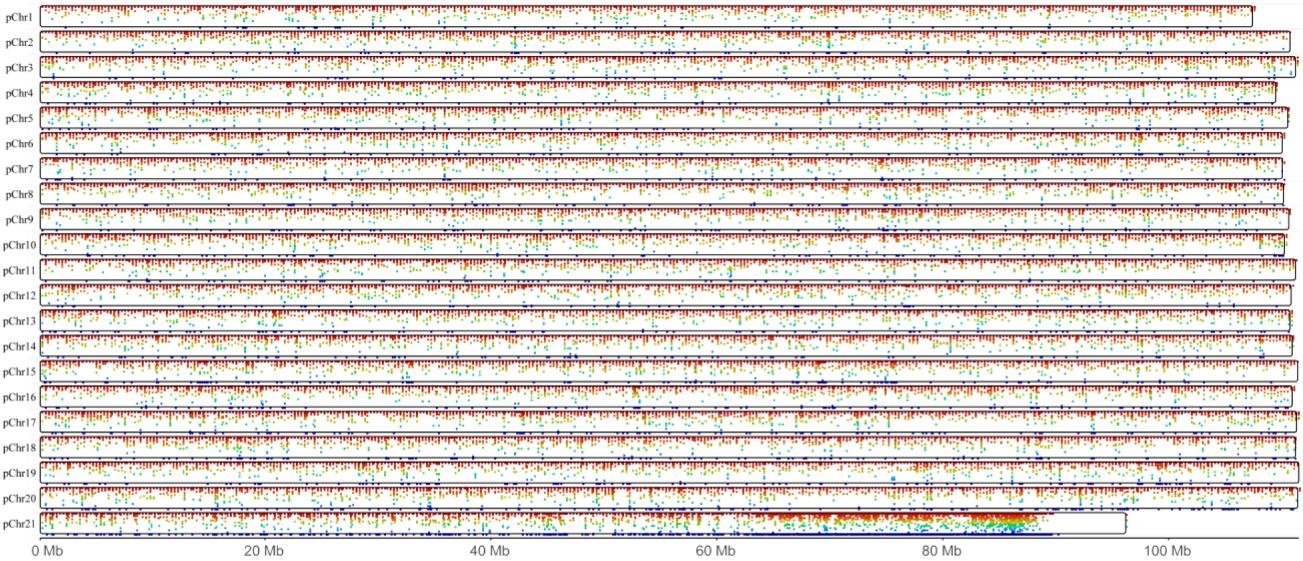

Supplement: Supplemental Information 9 — There are 21 pseudo chromosomes in total. The red point represents the relative higher mean methylation level, and the green represents the relative lower mean methylation level. [file peerj-07-7847-s009.jpg]

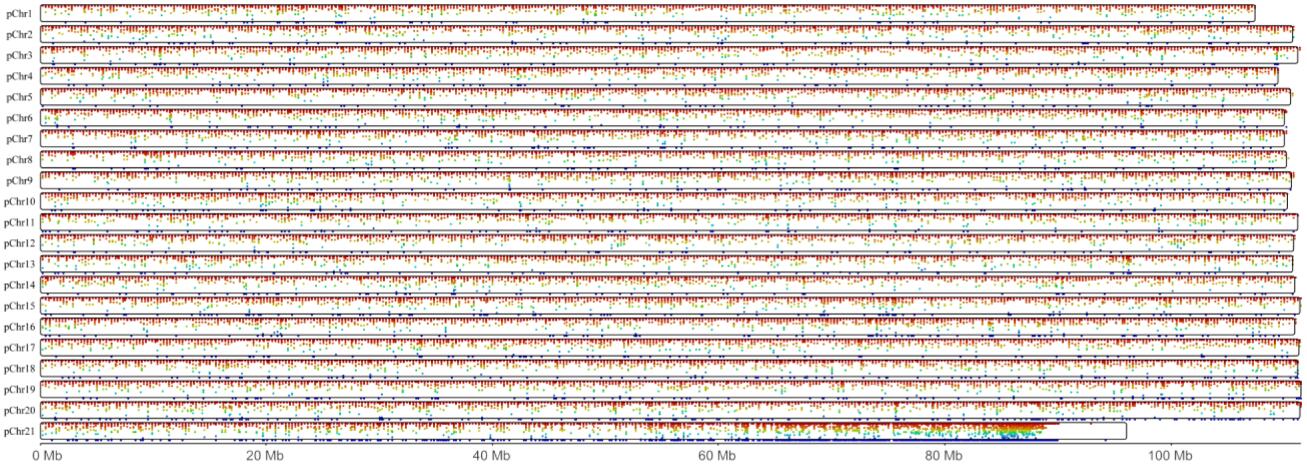

Supplement: Supplemental Information 10 — There are 21 pseudo chromosomes in total. The red point represents the relative higher mean methylation level, and the green represents the relative lower mean methylation level. [file peerj-07-7847-s010.jpg]

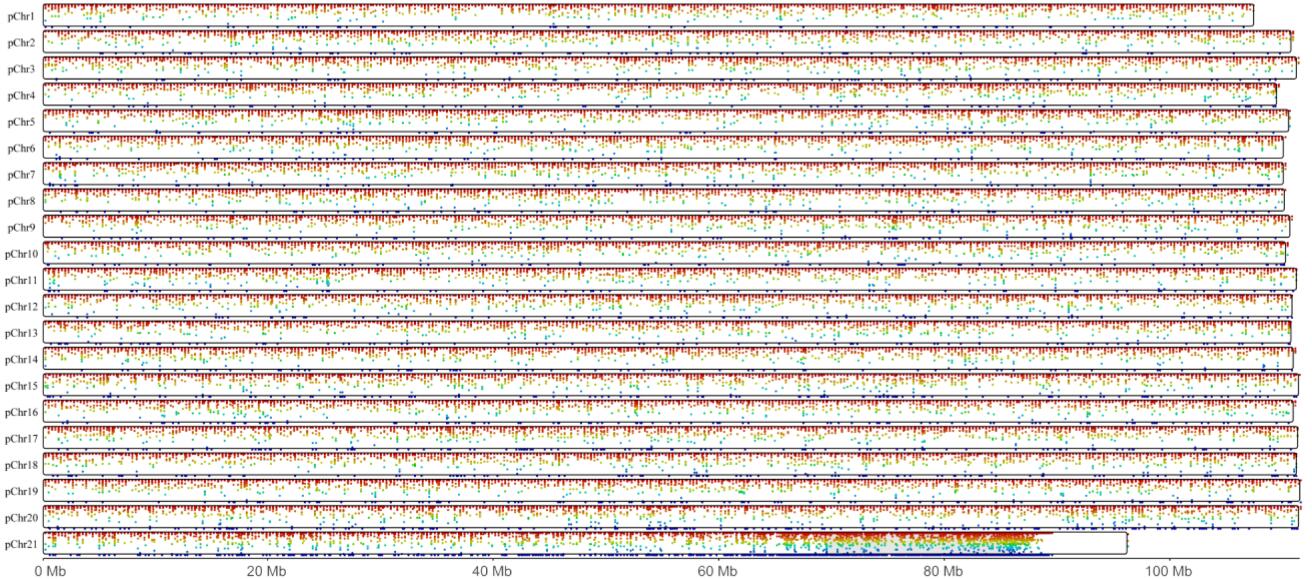

Supplement: Supplemental Information 11 — There are 21 pseudo chromosomes in total. The red point represents the relative higher mean methylation level, and the green represents the relative lower mean methylation level. [file peerj-07-7847-s011.jpg]

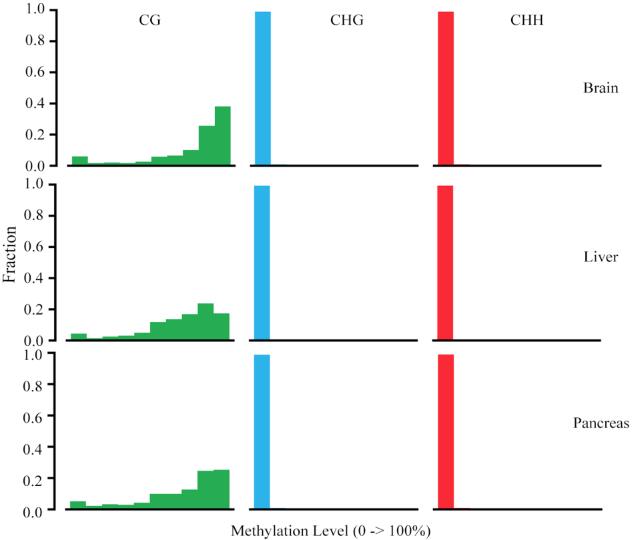

Supplement: Supplemental Information 12 — The X-axis represents mean methylation levels binned in 10 increment of 10% (i.e., 0–10%, 10–20%), y-axis is the corresponding bin’s fraction. [file peerj-07-7847-s012.jpg]

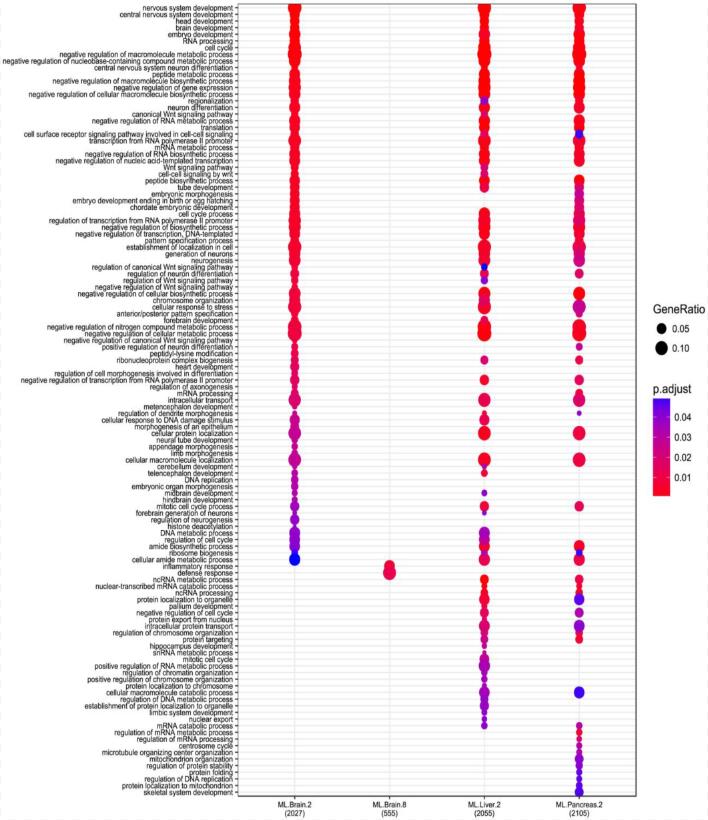

Supplement: Supplemental Information 13 — The X-axis is the LMP in brain, HMP in brain, LMP in liver and LMP in pancreas from left to right. The y-axis the GO items. [file peerj-07-7847-s013.jpg]

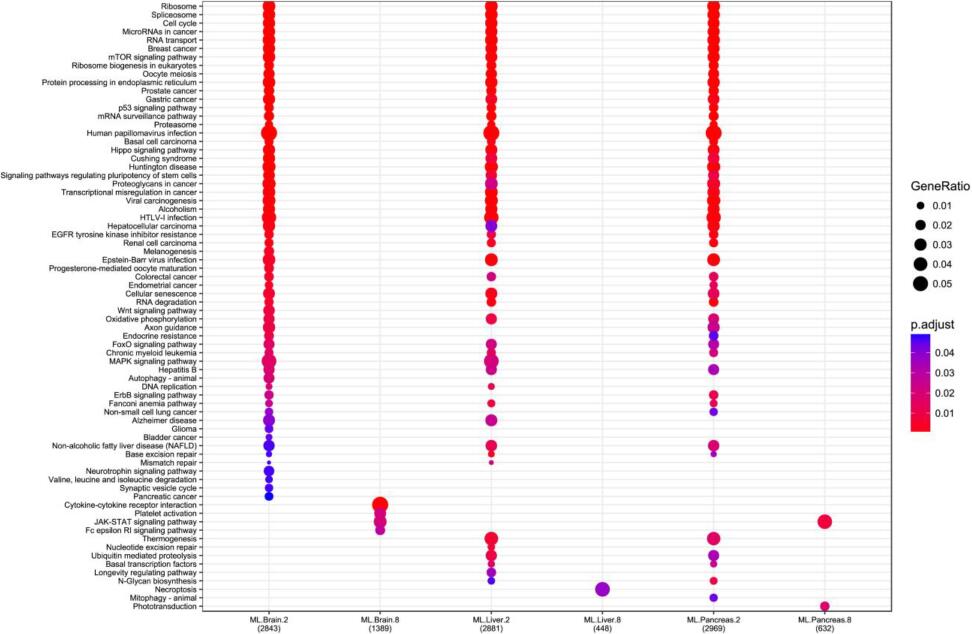

Supplement: Supplemental Information 14 — The X-axis is the LMP in brain, HMP in brain, LMP in liver, HMP in liver, LMP in pancreas and HMP in pancreas from left to right. The y-axis the KEGG pathways. [file peerj-07-7847-s014.jpg]

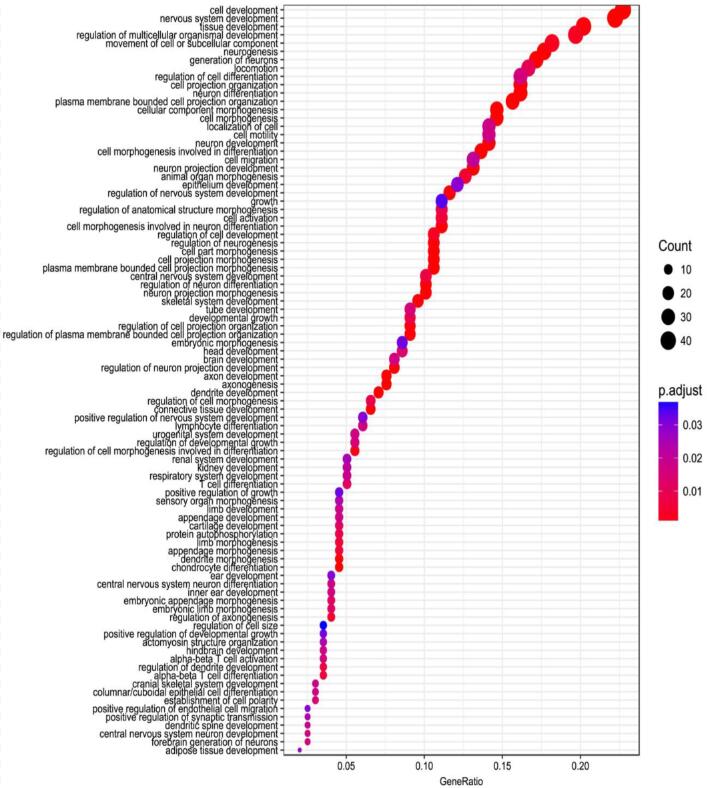

Supplement: Supplemental Information 15 — The X-axis is gene ratio and the y-axis is the enriched GO items. [file peerj-07-7847-s015.jpg]

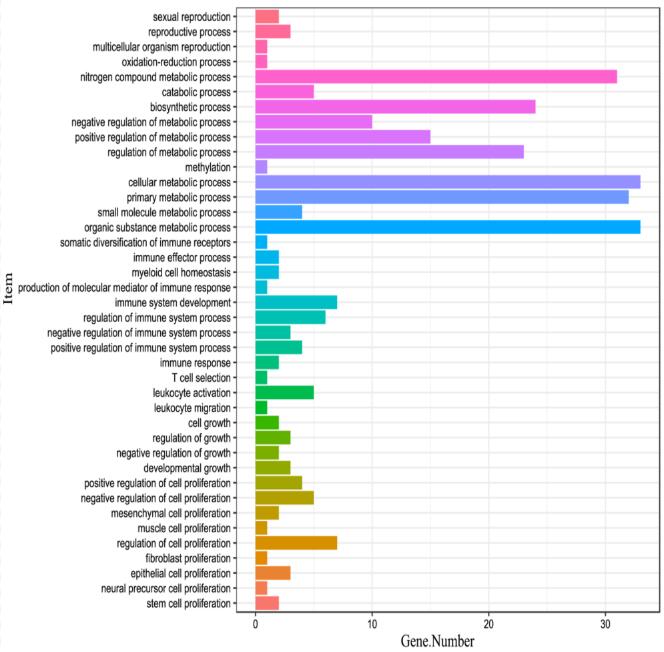

Supplement: Supplemental Information 16 — The X-axis is gene number and the y-axis is the enriched GO items. [file peerj-07-7847-s016.jpg]

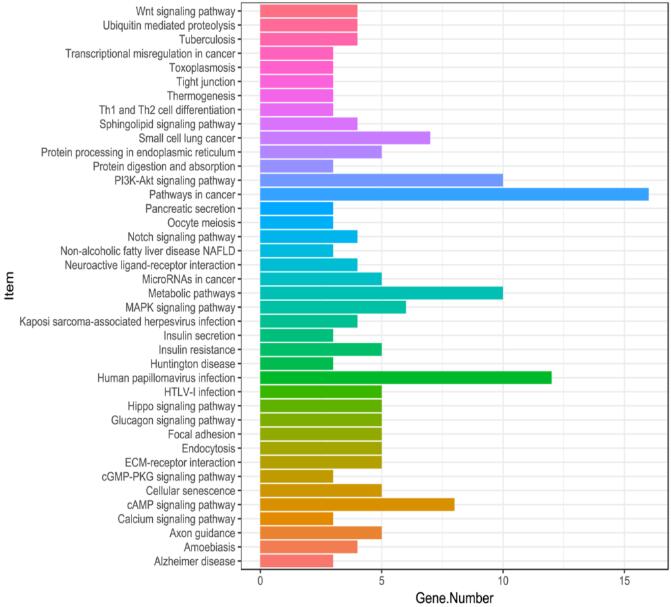

Supplement: Supplemental Information 17 — The X-axis is gene number and the y-axis is the enriched KEGG pathways. [file peerj-07-7847-s017.jpg]
